# In Vitro Evaluation of α-amylase and α-glucosidase Inhibition of 2,3-Epoxyprocyanidin C1 and Other Constituents from *Pterocarpus erinaceus* Poir

**DOI:** 10.3390/molecules28010126

**Published:** 2022-12-23

**Authors:** Romeo Toko Feunaing, Alfred Ngenge Tamfu, Abel Joel Yaya Gbaweng, Larissa Mekontso Magnibou, Fidele Ntchapda, Celine Henoumont, Sophie Laurent, Emmanuel Talla, Rodica Mihaela Dinica

**Affiliations:** 1 Department of Chemistry, Faculty of Sciences, University of Ngaoundere, Ngaoundere P.O. Box 454, Cameroon; 2 Department of Chemical Engineering, School of Chemical Engineering and Mineral Industries, University of Ngaoundere, Ngaoundere P.O. Box 454, Cameroon; 3 Department of Chemistry, Physics and Environment, Faculty of Sciences and Environment, Dunarea de Jos University, 47 Domneasca Str., 800008 Galati, Romania; 4 Department of Biological Sciences, Faculty of Science, University of Ngaoundere, Ngaoundere P.O. Box 454, Cameroon; 5 Laboratory of NMR and Molecular Imaging, Department of General, Organic Chemistry and Biomedical, University of Mons, B-7000 Mons, Belgium

**Keywords:** *Pterocarpus erinaceus*, 2,3-Epoxyprocyanidin C1, α-amylase inhibition, α-glucosidase inhibition, antidiabetic activity, phenolic compounds

## Abstract

Diabetes mellitus is a metabolic disorder which is one of the leading causes of mortality and morbidities in elderly humans. Chronic diabetes can lead to kidney failure, blindness, limb amputation, heart attack and stroke. Physical activity, healthy diets and medications can reduce the incidence of diabetes, so the search for more efficient antidiabetic therapies, most especially from natural products, is a necessity. Herein, extract from roots of the medicinal plant *Pterocarpus erinaceus* was purified by column chromatography and afforded ten compounds which were characterized by EIMS, HR-FAB-MS, 1D and 2D NMR techniques. Amongst them were, a new trimeric derivative of epicatechin, named 2,3-Epoxyprocyanidin C1 (1); two pentacyclic triterpenoids, friedelin (2) and betulin (3); angolensin (4); flavonoids such as 7-methoxygenistein (5), 7-methoxydaidzein (6), apigenin 7-O-glucoronide (8) and naringenin 7-O-β-D-glucopyranoside (9); and an ellagic acid derivative (10). The extract and compounds were evaluated for their antidiabetic potential by α-amylase and α-glucosidase inhibitory assays. IC_50_ values of compound **7** (48.1 ± 0.9 µg/mL), compound **8** (48.6 ± 0.1 µg/mL), compound **9** (50.2 ± 0.5 µg/mL) and extract (40.5 ± 0.8 µg/mL) when compared to that of acarbose (26.4 ± 0.3 µg/mL) indicated good α-amylase inhibition. In the α-glucosidase assay, the extract (IC_50_ = 31.2 ± 0.1 µg/mL), compound **7** (IC_50_ = 39.5 ± 1.2 µg/mL), compound **8** (IC_50_ = 40.9 ± 1.3 µg/mL), compound **1** (IC_50_ = 41.6 ± 1.0 µg/mL), Compound **4** (IC_50_ = 43.4 ± 0.5 µg/mL), compound **5** (IC_50_ = 47.6 ± 0.9 µg/mL), compound **6** (IC_50_ = 46.3 ± 0.2 µg/mL), compound **7** (IC_50_ = 45.0 ± 0.8 µg/mL), compound **9** (IC_50_ = 44.8 ± 0.6 µg/mL) and compound **11** (IC_50_ = 47.5 ± 0.4 µg/mL) all had moderate-to-good inhibitions, compared to acarbose (IC_50_ = 22.0 ± 0.5 µg/mL). The ability to inhibit α-amylase and α-glucosidase indicates that *P. erinaceus* and its compounds can lower blood glucose levels by delaying hydrolysis of carbohydrates into sugars, thereby providing a source of natural antidiabetic remedy.

## 1. Introduction

Diabetes mellitus is a deadly metabolic disease which results from excessive amounts of sugar in the blood of the patient, either due to insufficient production of insulin or inability of the cells to use the produced insulin properly [1]. Some factors, such as physical inactivity, obesity and aging increase the chances of diabetes and the prevalence is rising steadily from about 171 million affected persons worldwide in 2000, with an expectancy of more than 366 million patients by 2030, and will probably attain a peak of 693 million diabetic individuals worldwide by 2045 [2,3]. It is estimated that over 400 million people worldwide are exposed to the risk of developing type 2 diabetes mellitus (T2DM), which accounts for over 1.5 million annual deaths and also causes cardiovascular diseases, limb amputation, blindness, kidney failure and erectile disfunction; this can be controlled by healthy lifestyle and diet, non-smoking, normal body weight and physical activity [4,5]. Type 1 diabetes refers to the hyperglycemia resulting from a total absence of insulin secretion; meanwhile, T2DM consists of the resistance or inability to utilize insulin by the peripheral tissues and inadequate compensatory secretion of insulin; α-amylase and α-glucosidase inhibition is a suitable remedy to T2DM [6]. This is logical, since the dietary carbohydrates and polysaccharides are digested into sugars by enzymes of the alimentary canal such as pancreatic amylase and alpha glucosidase, creating an increase in the post-prandial glucose levels in blood [7]. It is normal that most metabolic diseases such as diabetes and others, especially those that are worsened by oxidative stress, are properly managed or treated by inhibiting the enzymes that are involved in their pathogenesis [8,9,10]. The use of modern drugs such as voglibose, miglitol, acarbose and pycnogenol to manage diabetes are usually associated with side effects such as diarrhea, flatulence, abdominal pain, bloating and discomfort [11]. This gives room for continuous search of herbal treatments from medicinal plants whose antidiabetic actions can be attributed to the phenolics, terpenoids, flavonoids and coumarin phytochemicals that they contain [12,13,14]. The management of the diabetic condition through the intake of conventional oral antidiabetics and insulin may be expensive and unaffordable to some patients and may also result in some side effects [15]. Over 400 medicinal plants have been described for antidiabetic activity; however, the search for new and more potent antidiabetic natural products continues to be attractive [16]. Recently, a number of new biologically active natural products have been isolated from medicinal plants and have exhibited antidiabetic properties, including α-amylase and α-glucosidase inhibitors, with greater efficacy than conventional oral hypoglycemic drugs, indicating that traditional medicine offers a bright future for diabetic therapies if they undergo good clinical practices [17,18]. In pharmacology, hundreds of compounds are being developed as antihyperglycemic agents, discovered from natural or synthetic sources and many of them are actually in clinical trial stages [19].

Most people, especially in the developing countries, recourse to traditional medicine for treatment of diabetes using medicinal plants. Pterocarpus belong to the class of Dalbergieae and consist of about 35 species, of mostly trees and woody climbers distributed in tropical regions, including *Pterocarpus erinaceus*, a medium-sized deciduous tree of about 12–15 m tall when fully grown [20]. *Pterocarpus erinaceus* is widely used in traditional medicine; for example, the decoction from its leaves has aphrodisiac effects and is also used to treat fever, intestinal wounds, headache, madness, malaria and syphilis [21]. The stem bark of this plant is used to treat inflammatory conditions such as ulcers, rheumatism, dermatitis and infections [22]. The roots are used to treat stomach aches, anemia, constipation, hemorrhoid and painful menstruation. Previous chemical investigations on *Pterocarpus erinaceus* report that this plant contains flavonoids, triterpenes and saponins [22,23,24]. The following classes of compounds isolated from *P. erinaceus*, including phenolic compounds and triterpenoids, are known to possess antidiabetic properties.

In a bid to contribute to the ongoing search for natural antidiabetic therapies, this study was designed to isolate and characterize bioactive secondary metabolites from the root extracts of *Pterocarpus erinaceus* and further evaluate their antidiabetic effects through inhibitory potential on α -amylase and α-glucosidase.

## 2. Results

### 2.1. Structural Elucidation of Compound ***1***

Compound **1** was obtained from the root extract of *Pterocarpus erinaceus*, in the form of a brownish powder in the eluent phase ethylacetate:methanol (90:10). This compound gave a positive Ferric test (FeCl_3_) and Shinoda test, indicating that it is a phenolic compound with flavonoid structure. The molecular formula of the compound was deduced from its high resolution FAB in positive mode which had the quasi-molecular ion [M + H]^+^ at m/z 865.1980, corresponding to the molecular formula C_45_H_36_O_18_, having 28 double bond equivalences. The IR spectrum of this compound had maxima at 3333.52 cm ^−1^, indicating the presence of hydroxyl groups between 1585 cm^−1^ and 1400 cm^−1^, corresponding to aromatic double bond vibrations and C-O bond stretching, which appeared within 1050 cm^−1^ and 1300 cm^−1^ range.

On the ^1^H NMR spectrum of compound, thirteen aromatic proton signals appeared and, based on their spin couplings, they were classified into different substitution patterns. Spin-spin coupling identified three aromatic rings with 1,3,4-trisubstitution, a first set at *δ*_H_ 7.18 ppm (d, *J* = 2.1 Hz), 7.02 ppm (d, *J* = 8.7 Hz), 7.18 ppm (dd, *J* = 2.1; 8.7 Hz); a second set at *δ*_H_ 6.73 ppm (d, *J* = 2.1 Hz), 6.83 ppm (d, *J* = 8.7 Hz), 6.84 ppm (dd, *J* = 2.0; 8.5 Hz); and a third set at *δ*_H_ 6.76 ppm (d, *J* = 2.0 Hz), 6.75 ppm (d, *J* = 8.5 Hz) and 6.73 ppm (dd, *J* = 2.0; 8.5 Hz). Another set of signals suggesting tetrasubstituted aromatic rings were observed at *δ*_H_ 5.95 ppm (d, *J* = 2.3 Hz), 6.00 (d, *J* = 2.3 Hz), together with two signals corresponding to two protons of pentasubstituted aromatic rings at *δ*_H_ 5.80 ppm (1H, s) and *δ*_H_ 6.09 ppm (1H, s). There were five signals of oxymethine protons which appeared at *δ*_H_ 5.69 ppm (d, *J* = 8.0 Hz), 4.38 ppm (d, *J* = 8.0 Hz), 4.11 ppm (d, *J* = 8.0 Hz), 4.38 ppm (m), 2.79 ppm (dd, *J* = 5.0; 16.0 Hz) and 2.40 ppm (dd, *J* = 8.0, 16.0 Hz), which are characteristic signals of a flavan-3-ol subunit in a 2,3-trans configuration [25]. The ^1^H NMR data given above suggests that the compound is a trimer, constituted of flavan subunits, since position 4 in subunit 1 and 2 are substituted, and serve as linkage points between the subunits, with the protons present each as a methine with signals of an AB system resonating at *δ*_H_ 4.55 ppm (1H, d) and 4.14 ppm (d, *J* = 3.5 Hz). Notably, the coupling constant, *J* = 3.5 Hz, is in conformity with a relative 3,4-trans configuration [26].

^1^H-^1^H correlations from the COSY spectrum presented important cross peaks between different pairs of adjacent protons as follows: The proton with signal *δ*_H_ 4.14 ppm (H-4C) and the proton at *δ*_H_ 3.28 ppm (H-3C); *δ*_H_ 5.69 ppm (H-3B) and the protons with signal at *δ*_H_ 4.12 ppm (H-2B) and *δ*_H_ 4.55 ppm (H-4B); the protons with signal at *δ*_H_ 3.85 ppm (H-3A) and *δ*_H_ 4.37 ppm (H-4A) and *δ*_H_ 2.82 (H-2A). Other cross peaks were observed on the COSY spectrum, indicating a correlation between the AB system of protons at *δ*_H_ 6.00 ppm (H-8C) and *δ*_H_ 5.95 ppm (H-6C), which characterises a terasubstituted benzene ring.

The ^13^C NMR spectrum broadband and DEPT spectra of compound **1** showed signals corresponding to a total of 45 carbon atoms, which substantiates the hypothesis of compound **1** being a flavonoid trimer. Classification of the forty-five carbon atom signals into quaternary carbon signals, methine and methylene carbon signals was facilated by ^13^C NMR and DEPT experiments. Characteristic signal of one methylene (CH_2_) carbon appeared at *δ*_C_ 28.9 (C-4A), and twenty methine (CH) carbons, out of which thirteen were aromatic methines, with signals at *δ*_C_ 121.4 (C-2′B), 119.9 (C-2′C), 119.4 (C-2′A), 116.7 (C-3′B), 116.1 (C-3′A), 116.0 (C-3′C), 115.7 (C-6′B), 115.7 (C-6′C), 115.5 (C-6′A), 98.3 (C-6C), 96.6 (C-8C), 96.4 (C-8A) and 96.1 (C-6B). Five of the methine carbons were oxygenated and their signals appeared at *δ*_C_ 80.3 (C-2A), 78.9 (C-3B), 72.6 (C-2B), 67.5 (C-3A) and 67.2 (C-3C). Signals of two sp3 methines were observed at *δ*_C_ 38.3 (C-4B) and 29.8 (C-4A), corresponding to the methines at the linkages between the units. Signals of twenty-four quaternary carbon atoms were observed, out of which twenty-three were aromatic quaternary carbons, whose signals appeared at *δ*_C_ 157.8 (C-7C), 156.7 (C-7C), 156.0 (C-7B), 155.8 (C-7A), 155.8 (C-5A), 155.5 (C-5B), 154.1 (C-9C), 151.8 (C-9B), 146.6 (C-4′C), 1463 (C-4′B), 145.9 (C-4′A), 145.8 (C-4′5), 145.5 (C-5′B), 145.3 (C-5′A), 133.2 (C-1′A), 132.5 (C-1′C), 131.8 (C-1′B), 108.9 (C-10B), 106.7 (C-8A), 106.4 (C-8B), 104.9 (C-10C) and 100.0 (C-10A). One non-aromatic quaternary carbon signal was observed at *δ*_C_ 99.9 (C-2C).

**Figure 1:**
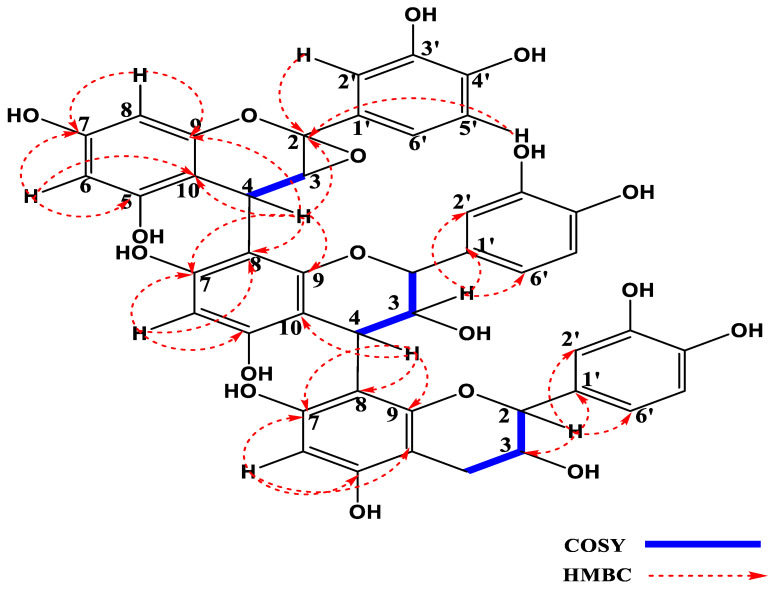
HMBC and COSY correlations of compound **1** (2,3-Epoxyprocyanidin C1).

HSQC (^1^J_C-H_) correlations were used to attribute each proton to its corresponding carbon atom and HMBC enabled the constitution of the molecule, which was similar to procyanidin C1 [26]. The conspicuous difference between compound **1** and procyanidin C1 is visible on the DEPT spectrum where five oxymethine signals appeared for compound **1**, instead of six for procyanidin C1, and the presence of a quaternary carbon atom at *δ*_C_ 99.9 (C-2C) in compound **1**, which is absent in procyanidin C1, suggests that one of the methines in procyanidin C1 is oxidized in compound **1**. From HMBC correlations, and by comparison with the chemical shifts of 2α,3α-epoxy-5,7,30,40-tetrahydroxyflavan-(4b-8-catechin), it is observed that there is an epoxide formed between the carbons C-2 and C-3 in the C unit, and a similar observation was made for catechin monomeric unit [25]. This epoxidation, as well as its position, is unambiguously attributed with the aid of HMBC correlations between the protons *δ*_H_ 7.02 ppm (H-2′C) and the carbon signals at *δ*_C_ 99.9 (C-2C)/132.5 (C-1′C) and 145.8 (C-3′C), as well as between the proton at *δ*_H_ 6.82 ppm (H-5′C) and the carbons at *δ*_C_ 99.9 (C-2C) and 146.6 (C-4′C). The HMBC analysis also facilitated the determination of the various linkages between the units A, B and C as indicated on Figure 1. The connection between unit A and B was confirmed by the HMBC correlations between the proton at *δ*_H_ 4.55 ppm (H-4B) and the carbon atoms with signals at *δ*_C_ 106.7 (C-8A)/151.1 (C-9A) and 155.8 (C-7A), while the junction between unit B and C was established through the HMBC correlations between the protons at *δ*_H_ 4.14 ppm (H-4C), with the carbon signals *δ*_C_ 106.4 (C-8B)/151.1 (C-9B)/156.0 (C-7C)/104.9 (C-10A) and 99.9 (C-2C). Other HMBC correlations were visible between the proton *δ*_H_ 4.55 (H-3B) and carbon signals *δ*_C_ 131.8 (C-1′B)/115.7 (C-2′B) and 121.4 (C-6′B) and correlations of the proton at *δ*_H_ 4.37 ppm (H-2A) and the carbon signals at *δ*_C_ 67.5 (C-3A)/133.2 (C-1′A)/115.5 (C-2′A) and 119.5 (C-6′A). Figure 1 below shows important HMBC and COSY correlations of compound **1**.

**Figure 2:**
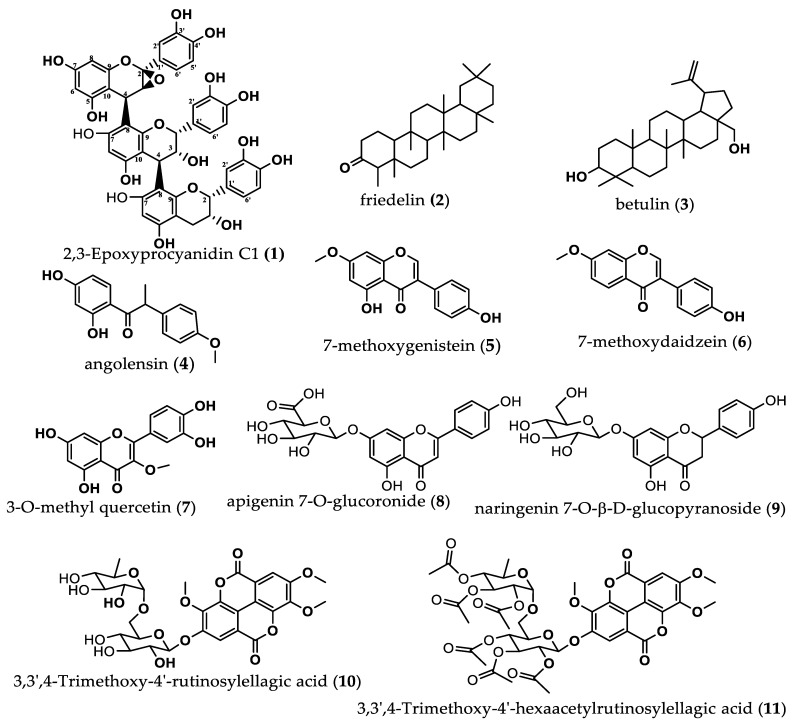
Structures of compounds isolated from roots of *Pterocarpus erinaceus*.

The set of above spectral data and the coupling constants of the different protons, notably 3.28 (1H, d, *J* = 3.4 Hz), 4.11 (1H, d, *J* = 2.0 Hz), 4.37 (1H, d, *J* = 8.5 Hz) and 2.82 (1H*α*, dd, *J =* 4.16 Hz), and by comparison with reported data for procyanidin C1, confirmed that compound **1** is a trimer made up of one unit of catechin and two units of epicatechin. The chemical shifts of this compound are given on Table 1. The connection of 2,3-*trans* catechin unit and 2,3-*cis* epicatechin unit is confirmed by the signals *δ*_C_ 80.3 ppm (C-2A) and 72.6 (C-2B) [26], as well as *δ*_C_ 67.2 (C-2C) [25]. The set of IR, mass and NMR spectra of compound **1**, to which the name 2,3-Epoxyprocyanidin C1 was given, are provided as Supplementary Material Figures S1–S9. The structures of all the compounds isolated from *P. erinaceus* and characterized are provided in Figure 2 below.

### 2.2. Antidiabetic Activity of Extract and Compounds

The enzymes α-amylase and α-glucosidase are responsible for the digestion of carbohydrates in the gastrointestinal tract, and the resulting monosaccharides are usually absorbed and, if in excess, diabetic conditions can arise. The reduction or delay in carbohydrate digestion and absorption is usually achieved through the inhibition of the carbohydrate digestive enzymes, α-amylases and α-glucosidases, and this is a suitable and effective means of reducing blood sugar levels and reducing the risk of developing diabetes and other metabolic diseases [27]. The extracted, isolated compounds, as well as the semi-synthetic derivative, compound **11**, were evaluated for their antidiabetic activity through the inhibition of α-amylase and α-glucosidase and the results reported on Table 2. The percentage of inhibitions at the highest test concentration of 50 µg/mL, as well as the concentrations with inhibition of 50% (IC_50_) where determined, were reported and indicated good antidiabetic potential. At 50 µg/mL, moderate percentage inhibition of α-amylase was observed for compound **1** (34.3 ± 0.7%), compound **4** (33.7 ± 0.5%), compound **5** (32.2 ± 1.0%), compound **6** (34.2 ± 0.5%), compound **10** (45.4 ± 0.3%) and compound **11** (43.5 ± 0.6%). The percentage inhibitions of other samples against α-amylase, such as compounds **7**, **8**, **9** and the extract were good at 50 µg/mL, as they were close to that of the standard acarbose used in the assay. The IC_50_ values of compound **7** (48.1 ± 0.9 µg/mL), compound **8** (48.6 ± 0.1 µg/mL), compound **9** (50.2 ± 0.5 µg/mL) and the extract (40.5 ± 0.8 µg/mL) compared to that of acarbose (26.4 ± 0.3 µg/mL) shows that these samples appreciably inhibited α-amylase within the tested concentrations. The α-amylase and α-glucosidase inhibitory potentials of compounds **2** and **3** were relatively low. For all samples, it was observed that α-glucosidase inhibitions were higher than α-amylase inhibitions. IC_50_ values of inhibition against α-glucosidase could be determined for all samples, except compound **2** and **3**, within the tested concentrations. The extract (IC_50_ = 31.2 ± 0.1 µg/mL) was the most active sample and its IC_50_ value was closest to that of acarbose (IC_50_ = 22.0 ± 0.5 µg/mL). The most active compounds were compound **7** (IC_50_ = 39.5 ± 1.2 µg/mL), compound **8** (IC_50_ = 40.9 ± 1.3 µg/mL) and compound **1** (IC_50_ = 41.6 ± 1.0 µg/mL). Compound **4** (IC_50_ = 43.4 ± 0.5 µg/mL), compound **5** (IC_50_ = 47.6 ± 0.9 µg/mL), compound **6** (IC_50_ = 46.3 ± 0.2 µg/mL), compound **7** (IC_50_ = 45.0 ± 0.8 µg/mL), compound **9** (IC_50_ = 44.8 ± 0.6 µg/mL) and compound **11** (IC_50_ = 47.5 ± 0.4 µg/mL) also had good inhibitory activity against α-glucosidase. It was observed that the extract had greater antidiabetic potential than the isolated compounds, and no tested sample was more active than the standard acarbose, but the activities of some of the samples were relatively good compared to that of acarbose. The α-amylase and α-glucosidase percentage inhibitions of the compounds and extract as well as IC_50_ values are given on Table 2 below.

## 3. Discussion

From the compounds isolated and characterized, it can be understood that this plant *P. erinaceus* contains pentacyclic triterpenoids, flavonoids and various phenolic compounds. Compound **1** was identified as a new trimer, a derivative of procyanidin C1, to which the name 2,3-Epoxyprocyanidin C1 was attributed. It should be noted that procyanidins or proanthocyanidins are plant polymeric phenols, made up from different amounts of flavan-3-ol monomers (catechin and epicatechin) with different structural diversities, resulting from differences in linkages and functional groups; they also have very important biological activities [28]. Compounds **2** and **3** were pentacyclic triterpenoids friedelin and botulin, respectively. Compound **4** was angolensin, while compounds **5** and **6** were two isoflavones, namely, 7-methoxygenistein and 7-methoxydaidzein, respectively. Compounds **7**, **8** and **9** were flavonoids, namely, 3-O-methyl quercetin, apigenin 7-O-glucoronide and naringenin 7-O-β-D-glucopyranoside, respectively. Compound **10** was identified as a derivative of ellagic acid called 3,3′,4-Trimethoxy-4′-rutinosylellagic acid, which was completely acetylated to compound **11**, identified as 3,3′,4-Trimethoxy-4′-hexaacetylrutinosylellagic acid. The class of compounds isolated and characterized in this study correlates with other studies reporting chemical constituents from this plant. Terpenoids, sterols and their glycosides have been described from this plant [23,29,30], as well as phenolics and flavonoids, which have been quantified in the extracts of this plant and also isolated [23,31,32].

*P. erinaceus* extracts, fractions and pure compounds possess bioactivities and have been used as remedies for some illnesses, including diabetes. The incidence of diabetes is rising at an alarming rate, despite the great amount of research that has been done in this domain to get proper medications that can help to lower blood sugar levels through various modes of action. The abnormal rise in blood sugar levels is usually associated with insufficiency in insulin production or reception, as well as insulin resistance, and secondary metabolites, including phenolic compounds, flavonoids and anthocyanidins, are known to possess antidiabetic properties and are able to reverse high blood glucose levels [33]. In this study, the extract from *P. erinaceus* exhibited great potential of lowering blood glucose levels, hence indicating that it can be used to manage type 2 diabetes mellitus (T2DM). The extract of this plant has been shown to exhibit hypoglycemic effects, and it was suggested that the mechanism could possibly be by decreasing glucose absorption and also by forming complexes with glucose molecules; the activity was attributed to the constituent phytochemicals which were terpenoids, saponins, flavonoids, alkaloids, tannins and other phenolic compounds present in the extract [34]. Phenolic-rich extracts are believed to possess good antidiabetic activities, acting by inhibiting the enzymes α-amylase and β-glucosidase, which are responsible for the hydrolysis of carbohydrates into glucose [35,36,37,38]. Inhibition of these enzymes can delay the breakdown and absorption of glucose in the blood, and is a suitable strategy for reducing blood glucose levels, thereby preventing the development of hyperglycemia and diabetes [39,40]. The compounds equally inhibited the carbohydrate digestive enzymes, but were less active compared to the crude extract; this suggests that the individual compounds have a synergistic effect in the crude extract. The compounds, when isolated, can be divided into terpenoids and phenolic compounds, and it was observed that the phenolic compounds were generally more active than the terpenoids.

Compound **1** (2,3-epoxyprocyanidin C1), a proanthocyanidin, inhibited α-amylase and β-glucisidase and natural inhibitors of amylase and glucosidase, are highly efficient antidiabetic remedies with little side effects. In one study, several proanthocyanidins having catechin and epicatechin monomers, ranging from trimers right up to undecamers, showed very good antidiabetic potential, and this correlated with antioxidant effects [35,41]. Procyanidins oligomers of catechin and epicatechin, as well as the monomers, were shown to suppress acute hyperglycemia by activating AMPK and insulin signaling pathways, and reduce postprandial blood glucose levels by glucose transporter 4 translocation [42]. The evidence of 2,3-epoxyprocyanidin C1 (compound **1**) to reduce hyperglycemia by inhibiting carbohydrate enzymes in this study supports increased interest in plant procyanidin extracts in treating diabetes. Procyanidin C1, as well as procyanidin-rich extracts from tea, grapes, cranberries, pine bark, cinnamon, apples, soybeans and cocoa have been reported to suppress hyperglycemia and obesity using rat model experiments [43,44,45,46,47,48]. Grape seeds are notably very rich in procyanidins and their regular consumption helps to maintain healthy glucose levels, as proven in experimental models [49,50,51]. Friedelin (compound **2**) exhibited low antidiabetic potential within the tested concentrations in this study, but nevertheless, with promising potential. Friedelin has been shown to possess antidiabetic potential at doses of 20 and 40 mg/kg, using STZ-induced diabetes in rat models [52]. Friedelin and some compounds of betulin type have been listed amongst antidiabetic phytochemicals [53]. The isoflavone derivatives, 7-methoxygenistein (compound **5**) and 7-methoxydaidzein (compound **6**), were able to inhibit both enzymes, α-amylase and α-glucosidase, almost to the same extent. It should be noted that these isoflavones are amongst the compounds with potent inhibitory action on carbohydrate digestive enzymes. This can be verified from a recent study which reported α-amylase and α-glucosidase inhibitory activities of similar compounds isolated from natural medicinal plants [54]. These compounds occur as antidiabetic soy isoflavones and exercise direct effects on β-cell proliferation, and stimulate insulin secretion, insulin uptake and action, besides their other beneficial effects as antioxidant, anti-apoptosis, estrogen receptor agonist and inhibition of tyrosine kinase, and equally preventing diabetic pathogenesis of muscles, liver, pancreas and adipose tissue [5,55,56,57,58,59,60]. These isoflavones, together with the other flavonoids, 3-O-methyl quercetin (compound **7**), apigenin 7-O-glucoronide (compound **8**) and naringenin 7-O-β-D-glucopyranoside (compound **9**), have been described as antidiabetic compounds capable of inhibiting carbohydrate enzymes [53,61,62,63,64,65]. It was further explained that, for flavonoids to exert antidiabetic and anti-inflammatory activities, a C2–C3 double bond on C-ring is necessary in addition to hydroxyl groups at C3′, C4′, C5, and C7 positions in rings A and B of a flavonoid basic skeleton, and a further substitution on C3 position enhances antidiabetic activity [66]. All the flavonoid compounds; 7-methoxygenistein (compound **5**), 7-methoxydaidzein (compound **6**), 3-O-methyl quercetin (compound **7**), apigenin 7-O-glucoronide (compound **8**) and naringenin 7-O-β-D-glucopyranoside (compound **9**), all possess a hydroxy group at either positions C3′, C4′, C5 or C7. Additionally, all of them except naringenin 7-O-β-D-glucopyranoside, have the C2–C3 double bond, satisfying the criteria for antidiabetic potential [66]. Flavonoids exhibit hypoglycemic activity by reducing the absorption of carbohydrates in the intestine, modulating the effects of glucose metabolic enzymes, enhancing insulin and β-cell functions [65]. This supports the α-amylase and α-glucosidase inhibition potential exhibited in this study by the phenolic compounds from *Pterocarpus erinaceus*.

The derivatives of ellagic acid, 3,3′,4-Trimethoxy-4′-rutinosylellagic acid (compound **10**) and 3,3′,4-Trimethoxy-4′-hexaacetylrutinosylellagic acid (compound **11**), equally exhibited antidiabetic potential by inhibiting both carbohydrate digestive enzymes, α-amylase and α-glucosidase. This could be obvious as some of the ellagic acid derivatives, as well as ellagic acid, have been investigated for their antidiabetic activities and results were positive. Ellagic acid and 3,3′,4-trimethoxyellagic acid from *Anogeissus latifolia* and *Eugenia jambolana* have been described to possess abilities in increasing insulin levels, improving β-cell function and reducing glycosylated hemoglobin and plasma glucose levels [53,67,68]. The enzyme α-amylase in saliva and pancreatic secretions in animals hydrolyze polysaccharides into monosaccharides such as glucose, while α-glucosidase in the small intestine breaks glycoside bonds in β-D-glucosides and oligosaccharides to produce glucose [17]. Since these two enzymes can greatly contribute to the rise in blood glucose levels in diabetic patients, as a result of carbohydrate digestion, their inhibition is a key step in the treatment of diabetes, especially T2DM. Inhibition of carbohydrate-hydrolyzing enzymes is a common therapeutic approach used in reducing postprandial hyperglycemia, thereby treating non-insulin-dependent diabetes (T2DM) and many marketed drugs and potential antidiabetic drug candidates fall in this category [69,70]. The results of this study therefore reveal a valuable potential for some of the isolated compounds and the extract of *P. erinaceus* as a source of molecules that can be used in the treatment of diabetes and for the development of antidiabetic drugs.

## 4. Materials and Methods

### 4.1. Plant Material

The roots of *Pterocarpus erinaceus* Poir. were collected during the month of July 2018 from Wack locality, in the Ngan-ha subdivision of the Adamawa Region of Cameroon. A voucher specimen of this plant was prepared and identified under the voucher number N° 5205/SRF.Cam., by Mr. Nana, a botanist working at the National Herbarium of Cameroon.

### 4.2. Extraction and Isolation

The roots of the *Pterocarpus erinaceus* were cut into pieces, dried under a shade for 5 weeks and at room temperature before being powdered. 2 kg of the resulting powder was extracted with 20 L CH_2_Cl_2_-MeOH (1:1) by maceration at room temperature. The supernatants were filtered and concentrated on a Rotary evaporator to give a crude extract. The extract (50 kg) was purified by column chromatography, using silica gel and an eluent gradient system *n*-hexane/AcOEt (100:0 to 0:100, *v/v*), followed by AcOEt/CH_3_OH (100:0 to 0:100, *v/v*), by order of increasing polarity to afford five fractions (A–E) based on TLC profiles.

Fraction A (3.0 g) was eluted on a silica gel column on a gradient eluent system *n*-hexane/AcOEt (100:0 to 50:50, *v/v*) and afforded two triterpenoids, compound **2** (12.0 mg) and compound **3** (21.5 mg). Purified on a silica gel column with *n*-hexane/AcOEt (80:20, *v/v*) isocratic eluent, fraction B (11.4 g) yielded three compounds, all in the form of yellowish solids as follows: compound **4** (37.0 mg), **5** (20.0 mg) and **6** (46.1 mg). Compound **7** (12.8 mg) was obtained in the form of yellow powder from fraction C (300 mg) by column chromatography, with the isocratic eluent system *n*-hexane/AcOEt (15:85, *v/v*). Fraction D (6.0 g) was rechromatographed on a silica gel column, with the gradient system AcOEt/CH_3_OH (100-0), and three compounds were obtained as follows: compound **10** (60.5 mg) as white crystals, compound **8** (23.4 mg) as yellow powder and compound **9** (12.0 mg) as yellow powder. Compound **1** (14.9 mg) was obtained from fraction E (3.0 g) as a dirty white powder through silica gel column chromatography AcOEt/CH_3_OH (80:20, *v/v*) isocratic system and further purified on Sephadex LH-20 eluted with pure MeOH. Part of compound **10** (20.0 mg) was subjected to acetylation to obtain compound **11** (15.0 mg), a hexaacetylated derivative of compound **10**.

### 4.3. Evaluation of Antidiabetic Activity by In Vitro α-amylase and α-glucosidase Inhibitory Assay

The α-amylase inhibitory activity was evaluated by using the starch– iodine method with some modifications [8,71]. The enzyme α-amylase from Bacillus licheniformis was used and enzyme solution was prepared with phosphate buffer (20 mM pH = 6.9 phosphate buffer prepared with 6 mM NaCl); then, 50 µL of α-amylase and 25 µL of different concentrations of compound or extract solutions were mixed in a 96-well microplate. The mixture was pre-incubated for 10 min at 37 °C; then, 50 µL of starch solution (0.05%) was added and incubated for 10 min at 37 °C. Following incubation, the reaction was completed by adding HCl (0.1 M, 25 µL) and Lugol (100 µL) solutions, and the absorbance was recorded at 565 nm.

The α-glucosidase inhibitory activity was evaluated according to the method described previously [72]. 50 µL of phosphate buffer (0.01 M pH 6.9), 10 µL of different concentrations of compound or extract solution, 50 µL of α-glucosidase from Saccharomyces cerevisiae in phosphate buffer (0.01 M pH 6.0) and PNPG (4-N-nitrophenyl-α-D-glucopyranoside) 25 µL in phosphate buffer (0.01 M pH 6.9) were mixed in a 96-well microplate. The solution was then incubated for 20 min at 37 °C.

Acarbose was used as a standard compound for both analyses. Results were given as percentage inhibition (%) at 100 µg/mL and 50% inhibition concentration (IC_50_).

## 5. Conclusions

High blood sugar levels, or hyperglycemia, results in the metabolic disease, diabetes mellitus, usually involving symptoms like increased hunger, thirst and urination. It leads to serious and fatal health complications, such as kidney failure, heart disease, limb ulcers, etc. Healthy eating habits and physical exercise, as well as insulin intake and other drugs are used to maintain normal blood glucose levels. However, since there is not a direct cure, natural therapies are equally being developed as an urgent response to the rising diabetic cases and shortcomings of conventional drugs. Natural α-glucosidase and α-amylase inhibitors are suitable candidates which can slow down or delay carbohydrate breakdown into sugars which will increase blood sugar levels. In this study, *Pterocarpus erinaceus* extract was purified by column chromatography and afforded ten compounds and one semi-synthetic derivative, which were characterized by extensive NMR and MS techniques. 2,3-Epoxyprocyanidin C1 was elucidated as a new derivative. The extract and compounds showed good potential inhibitory effects against α-amylase and α-glucosidase, indicating that they can reduce postprandial blood glucose levels and prevent diabetic conditions. The compounds 3-O-methyl quercetin, apigenin 7-O-glucoronide and naringenin 7-O-β-D-glucopyranoside, together with the extract, were the most active samples. This study ascertains the use of this plant in traditional medicine and in the management of diabetes-related health problems.

## Figures and Tables

**Table 1:** Spectral data of compound **1**: ^1^H NMR and ^13^C NMR with key HMBC and COSY.

Units	Position	^13^C(ppm)	^1^H(ppm)	HMBC	COSY
Unit A	2A	80.3	4.37 (1H, d, J = 8.5 Hz)	C2′B; C1′B; C6′B	H-2A/H-3A
	3A	67.5	3.85 (1H, t)	C10A	H-3A/H-2A; H-3A/H-4A
	4A	29.8	2.82 (1H*α*, dd, *J =* 4.16 Hz); 2.83 (1H*β*, brs)	C-2A; C-5A; C-10A; C-10A; C-3A	H-4A/H3A
	5A	155.8	-		
	6A	96.5	6.90 (1H, s)	C-10A; C-8A; C-7A	
	7A	155.8	-		
	8A	106.7	-		
	9A	151.1	-		
	10A	100.0	-		
	1′A	133.2	-		
	2′A	115.5	6.82 (1H, d, J = 8.9 Hz)	C-1′A; C-3′A	H-2′A/H-6′A
	3′A	145.3	-		
	4′A	145.9	-		
	5′A	116.1	6.75 (1H, d, J = 2.4 Hz)	C-1′A; C-4′A	H-5′A/H-2′A
	6′A	119.5	6.73(1H, dd, J = 8.9; 2.4 Hz)	C-2A; C-2′A; C-3′A	H-6′A/H-2′A; H-6′A/H-5′A
Unit B	2B	72.6	4,11 (1H, d, J = 2,0 Hz)		H-2B/H-3B
	3B	78.9	5.69 (1H, brs)	C-1′B; C-2′B; C-6′B	H-3B/H-2B; H-3B/H4-B
	4B	38.3	4.55 (1H, brs)	C-10B; C-7A; C-8A; C-9A	H-4B/H3B
	5B	155.5	-		
	6B	96.1	5.80 (1H, brs)	C-10B; C-7B; C-9B	
	7B	156.0	-		
	8B	106.4	-		
	9B	151.8	-		
	10B	108.9	-		
	1′B	131.8	-		
	2′B	115.7	6.63 (1H, d, *J* = 2.1 Hz)	C-1′BA; C-3′B	H-2′B/H-6′B
	3′B	145.5	-		
	4′B	146.3	-		
	5′B	116.7	7.30 (1H, d, *J* = 8.3 Hz)	C-1′B	H-5′B/H-2′B
	6′B	121.4	7.18 (1H, dd, *J* = 8.3, 21 Hz)	C-2B; C-3′A	H-6′B/H-2′B; H-6′B/H-5′B
Unit C	2C	99.9	-		
	3C	67.2	3.28 (1H, d, *J* = 3.4 Hz)	C-2A; C-9A	H-3C/H-4C
	4C	28.9	4.14 (1H, d, *J* = 3.4 Hz)	C-7B; C-8B; C-9B; C-10C; C-9C; C-2C	H-4C/H-3C
	5C	156.7	-		
	6C	98.3	5.95 (1H, d, *J* = 3.4 Hz)	C-10C; C-7C; C-8C	H-6C/H-8C
	7C	157.8	-		
	8C	96.6	6.00 (1H, d, *J* = 3.3 Hz)	C-10C; C-7C; C-6C	H-8C/H-6C
	9C	154.2	-		
	10C	104.9	-		
	1′C	132.5	-		
	2′C	115.7	7.02 (1H, d, *J* = 2.0 Hz)	C-2C; C-3′C	H-2′C/H-6′C
	3′C	145.8	-		
	4′C	146.6	-		
	5′C	116.0	6.75 (1H, d, *J* = 8.3 Hz)	C-1′C; C-4′C	H-5′C/H-2′C
	6′C	119.9	6.84 (1H, dd, *J* = 8.3, 2.1 Hz)	C-2C; C-1′C	H-6′C/H-2′C; H-6′C/H-5′C

**Table 2:** Antidiabetic activity (α-amylase and α-glucosidase inhibition).

Sample	*α*-amylase (% inh. at 50 µg/mL)	IC_50_ µg/mL	*α*-glucosidase (% inh. at 50 µg/mL)	IC_50_ µg/mL
1	34.3 ± 0.7	>50	53.4 ± 1.6	41.6 ± 1.0
2	21.5 ± 0.6	>50	32.1 ± 0.1	>50
3	19.3 ± 0.4	>50	28.5 ± 0.3	>50
4	33.7 ± 0.5	>50	54.1 ± 1.3	43.4 ± 0.5
5	32.2 ± 1.0	>50	50.6 ± 1.2	47.6 ± 0.9
6	34.2 ± 0.5	>50	51.8 ± 0.9	46.3 ± 0.2
7	50.2 ± 2.0	48.1 ± 0.9	54.0 ± 1.0	39.5 ± 1.2
8	50.9 ± 0.8	48.6 ± 0.1	55.2 ± 1.1	40.9 ± 1.3
9	50.5 ± 1.2	50.2 ± 0.5	53.7 ± 0.4	44.8 ± 0.6
10	45.4 ± 0.3	>50	51.8 ± 0.9	45.0 ± 0.8
11	43.5 ± 0.6	>50	50.7 ± 0.2	47.5 ± 0.4
Extract	53.2 ± 1.4	40.5 ± 0.8	66.9 ± 2.0	31.2 ± 0.1
Acarbose	72.5 ± 1.5	26.4 ± 0.3	81.7 ± 0.8	22.0 ± 0.5

## Data Availability

The data supporting reported results can be obtained from the corre.

## Supplementary Materials

The following supporting information can be downloaded at: https://www.mdpi.com/article/10.3390/molecules28010126/s1, Figure S1: HR-FAB (+) spectra of compound **1**; Figure S2. ESI-MS (+) spectra of compound **1**; Figure S3. IR spectrum of compound **1**; Figure S4: ^1^H-NMR spectrum (MeOD, 600 MHz) of compound **1**; Figure S5: ^13^C-NMR broadband spectrum (MeOD, 150 MHz) of compound **1**; Figure S6: ^13^C-NMR and DEPT-135 spectra (150 MHz, MeOD) of compound **1**; Figure S7: COSY ^1^H-^1^H spectrum of compound **1**; Figure S8: HSQC spectrum of compound **1**; Figure S9: HMBC spectrum of compound **1**.

## Sample Availability

Samples of some of the compounds are available from the authors.
