# Supplementary material for: In Vitro Evaluation of α-amylase and α-glucosidase Inhibition of 2,3-Epoxyprocyanidin C1 and Other Constituents from Pterocarpus erinaceus Poir
Source: Molecules. 2022 Dec 23;28(1):126. doi: 10.3390/molecules28010126 (PMC9822058; doi:10.3390/molecules28010126)
Supplement: Supplementary file 1 [file molecules-28-00126-s001.zip › molecules-2109705-supplementary.pdf]

molecules

Supplementary material

In vitro evaluation of α-amylase and α-glucosidase inhibition of 2,3-Epoxyprocyanidin C1 and other constituents from *Pterocarpus erinaceus* Poir

Romeo Toko Feunaing [1], Alfred Ngenge Tamfu [2,5*], Abel Joel Yaya Gbaweng [1], Larissa Mekontso Magnibou [1], Fidele Ntchapda [3], Celine Henoumont [4], Sophie Laurent [4], Emmanuel Talla [1,2], Rodica Mihaela Dinica [5*]

1 Department of Chemistry, Faculty of Sciences, University of Ngaoundere, P.O. Box 454 Ngaoundere, Cameroon
2 Department of Chemical Engineering, School of Chemical Engineering and Mineral Industries, University of Ngaoundere, P.O. Box 454 Ngaoundere, Cameroon
3 Department of Biological Sciences, Faculty of Science, University of Ngaoundere, P.O. Box 454 Ngaoundere, Cameroon
4 Laboratory of NMR and Molecular Imaging, Department of General, Organic Chemistry and Biomedical, University of Mons, B- 7000, Mons, Belgium
5 Department of Chemistry, Physics and Environment, Faculty of Sciences and Environment, 'Dunarea de Jos University', Galati, 47 Domneasca Str., 800008 Galati, Romania
* Correspondence: macntamfu@yahoo.co.uk (A.N.T.); rodica.dinica@ugal.ro (R.M.D.); Tel.: +237-675590353 (A.N.T.); +33-6130-251 (R.M.D.)

Table of Contents

- **Figure S1. HR-FAB (+) spectra of compound 1**
- **Figure S2. ESI-MS (+) spectra of compound 1**
- **Figure S3. IR spectrum of compound 1**
- **Figure S4. ^{1}H-NMR spectrum (MeOD, 600 MHz) of compound 1**
- **Figure S5. ^{13}C-NMR spectrum (MeOD, 150 MHz) of compound 1**
- **Figure S6. ^{13}C-NMR and DEPT-135 spectra (150 MHz, MeOD) of compound 1**
- **Figure S7. COSY ^{1}H-^{1}H spectrum of compound 1**
- **Figure S8. HSQC spectrum of compound 1**
- **Figure S9. HMBC spectrum of compound 1**

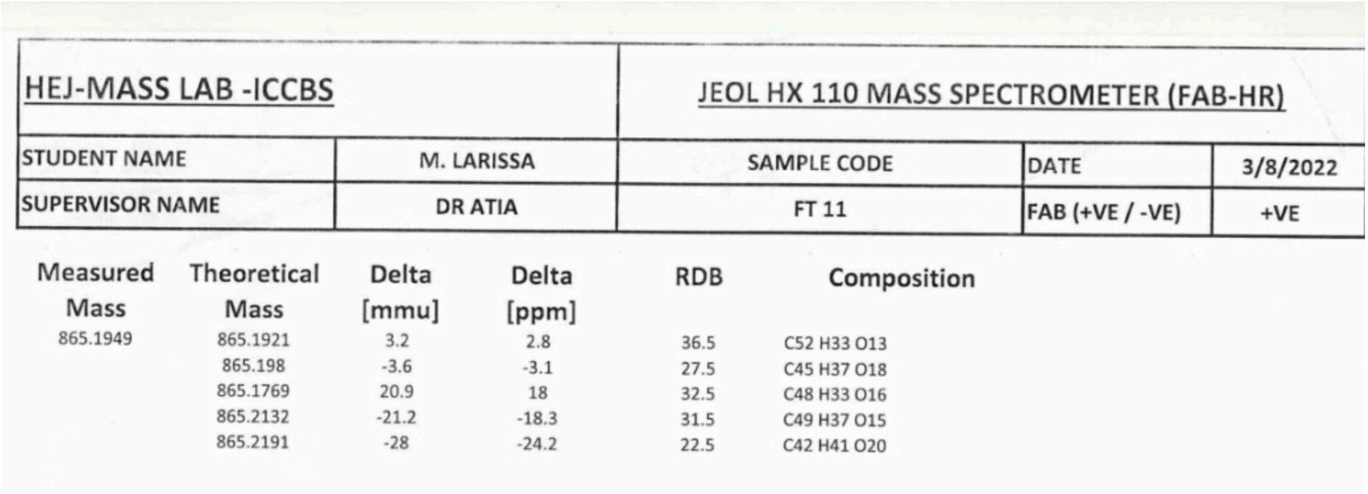

HEJ-MASS LAB -ICCBS		JEOL HX 110 MASS SPECTROMETER (FAB-HR)		
STUDENT NAME	M. LARISSA	SAMPLE CODE	DATE	3/8/2022
SUPERVISOR NAME	DR ATIA	FT 11	FAB (+VE / -VE)	+VE

Measured Mass	Theoretical Mass	Delta [mmu]	Delta [ppm]	RDB	Composition
865.1949	865.1921	3.2	2.8	36.5	C52 H33 O13
	865.198	-3.6	-3.1	27.5	C45 H37 O18
	865.1769	20.9	18	32.5	C48 H33 O16
	865.2132	-21.2	-18.3	31.5	C49 H37 O15
	865.2191	-28	-24.2	22.5	C42 H41 O20

Figure S1. HR-FAB (+) spectral window of compound 1

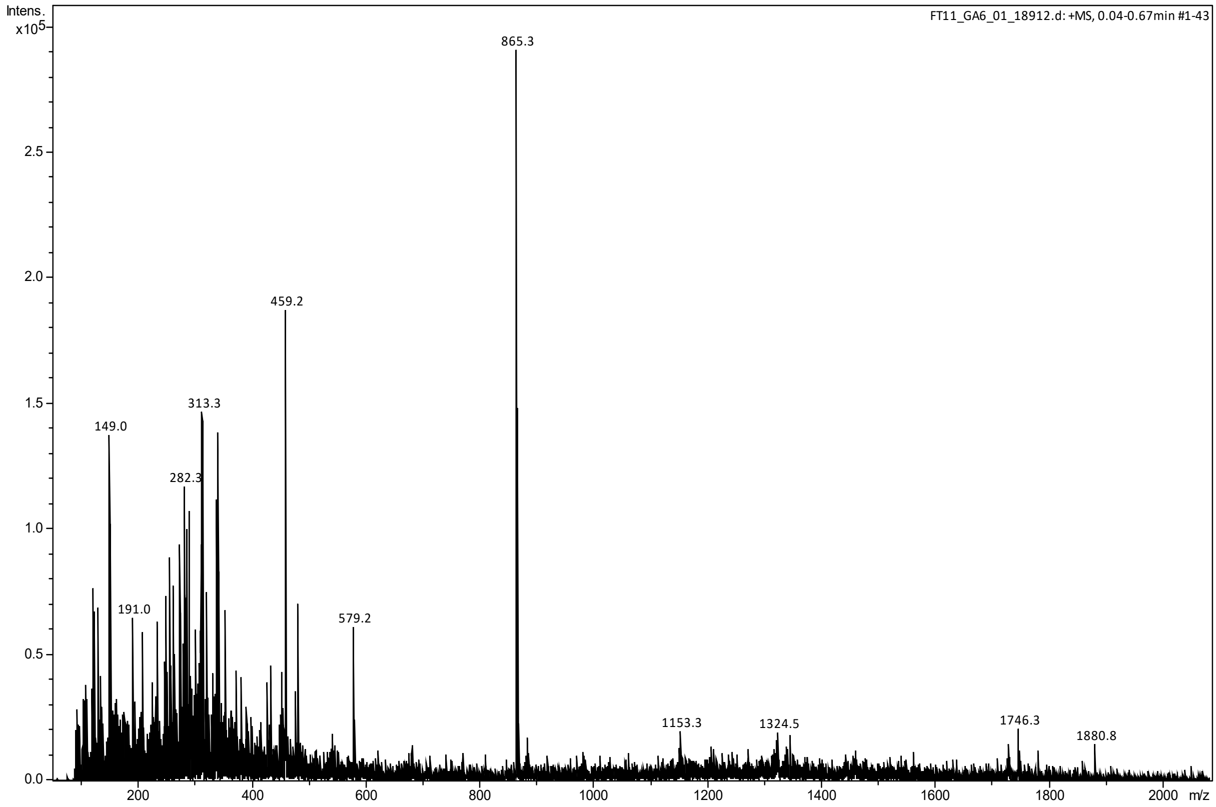

Figure S2. ESI-MS (+) spectra of compound 1

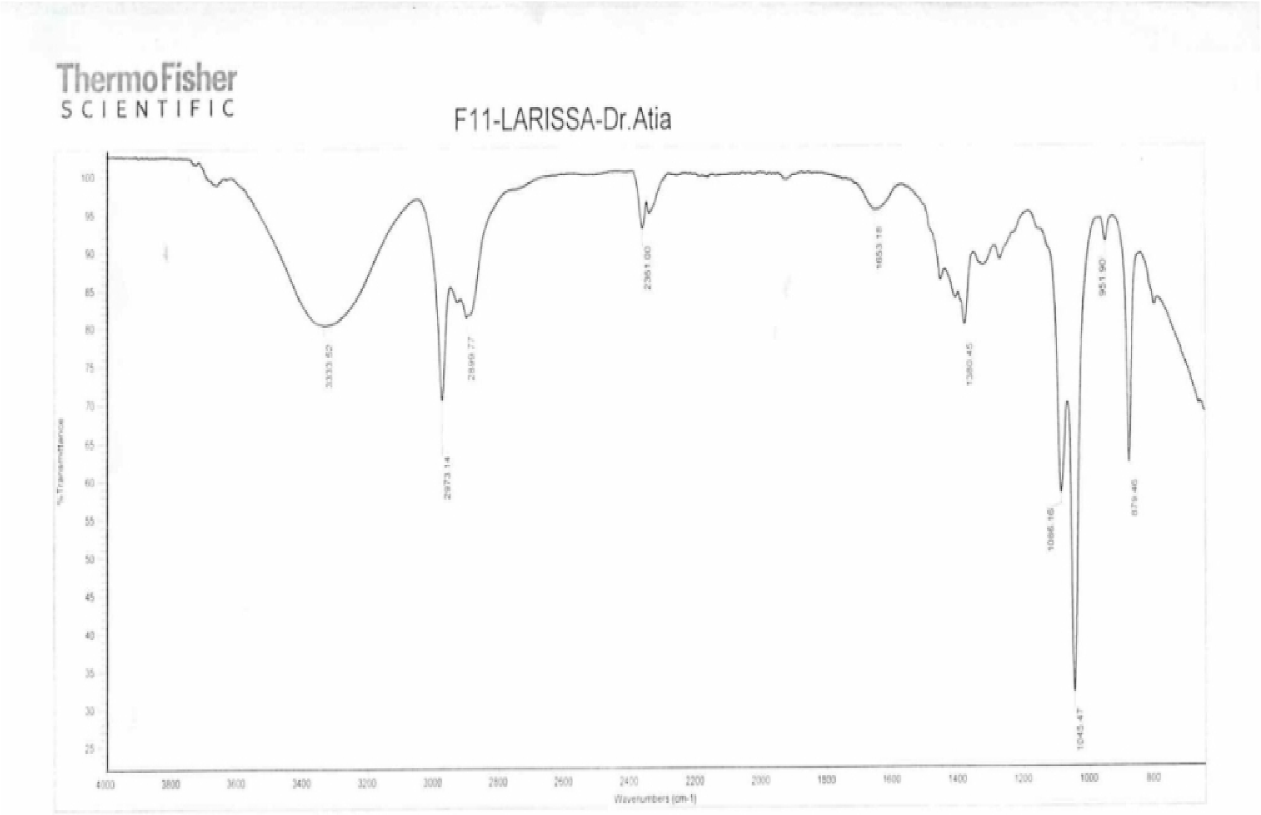

Figure S3. IR spectrum of compound 1

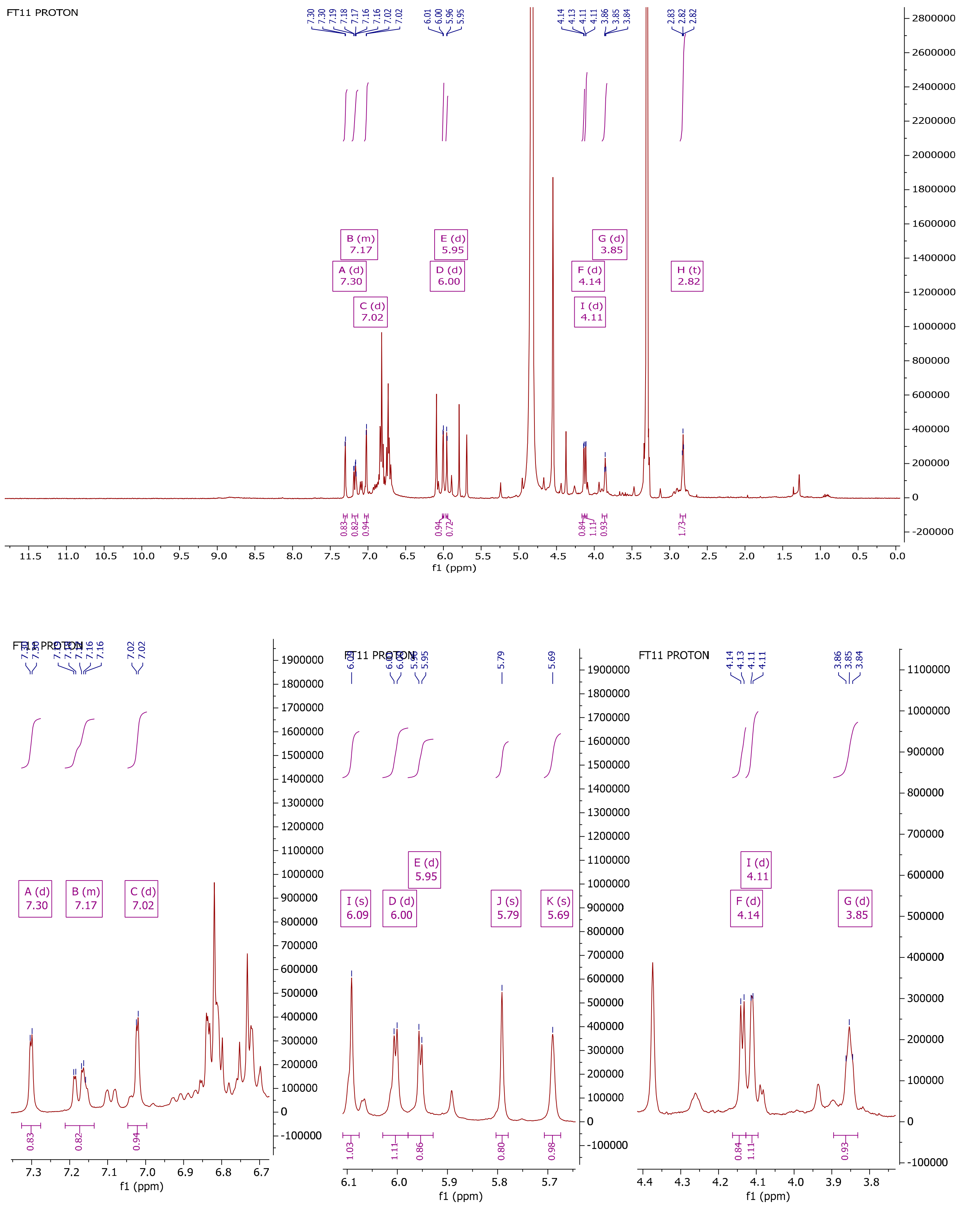

Figure S4. ^{1}H-NMR spectrum (MeOD, 600 MHz) of compound 1

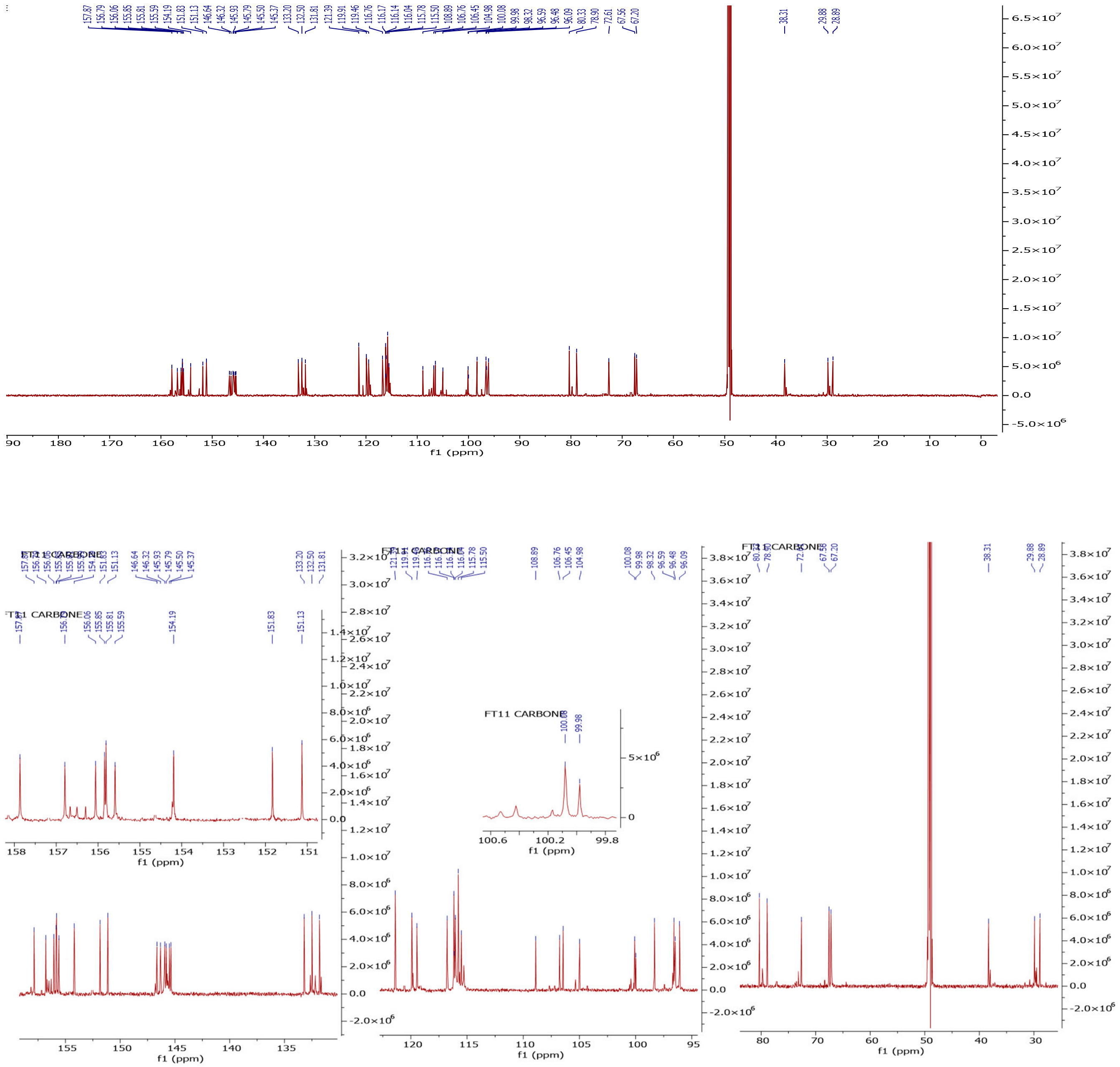

Figure S5. ^{13}C-NMR spectrum (MeOD, 150 MHz) of compound 1

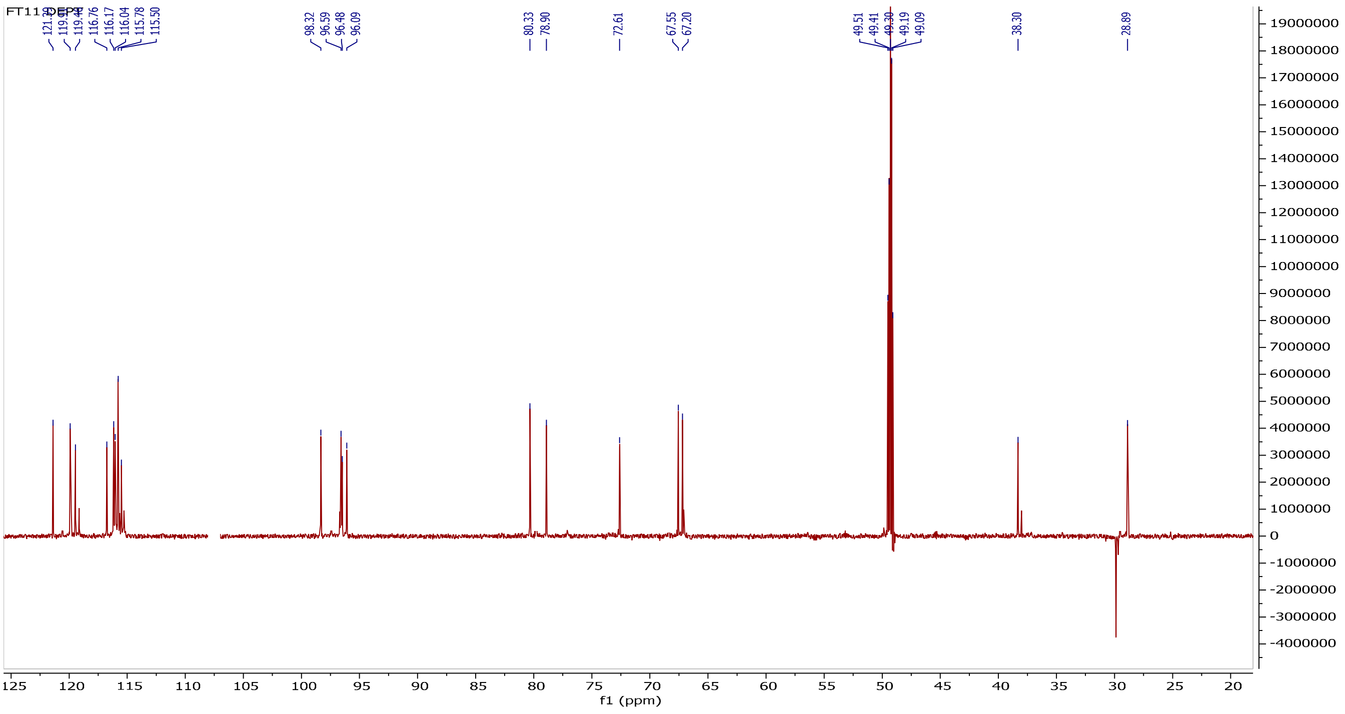

Figure S6. **^{13}C-NMR and DEPT-135 spectra (150 MHz, MeOD) of compound 1**

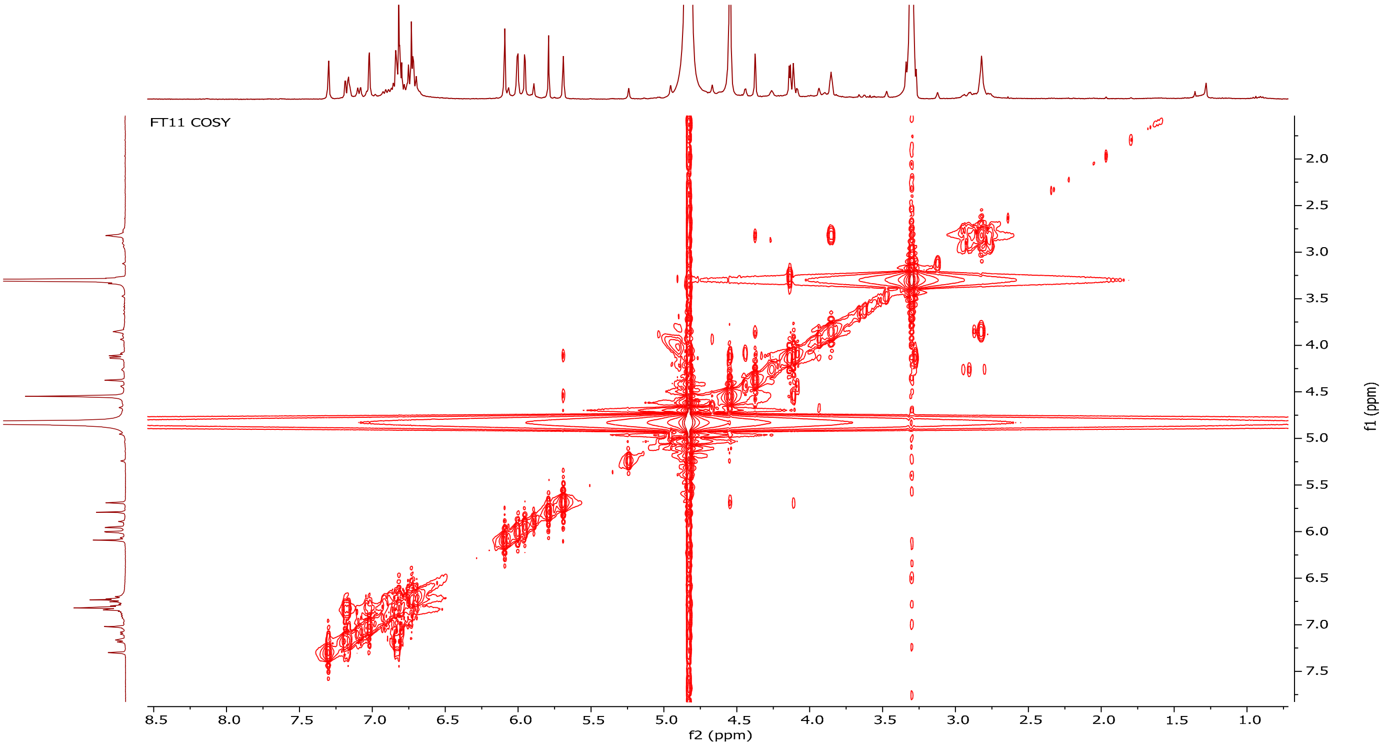

Figure S7. COSY ^{1}H-^{1}H spectrum of compound 1

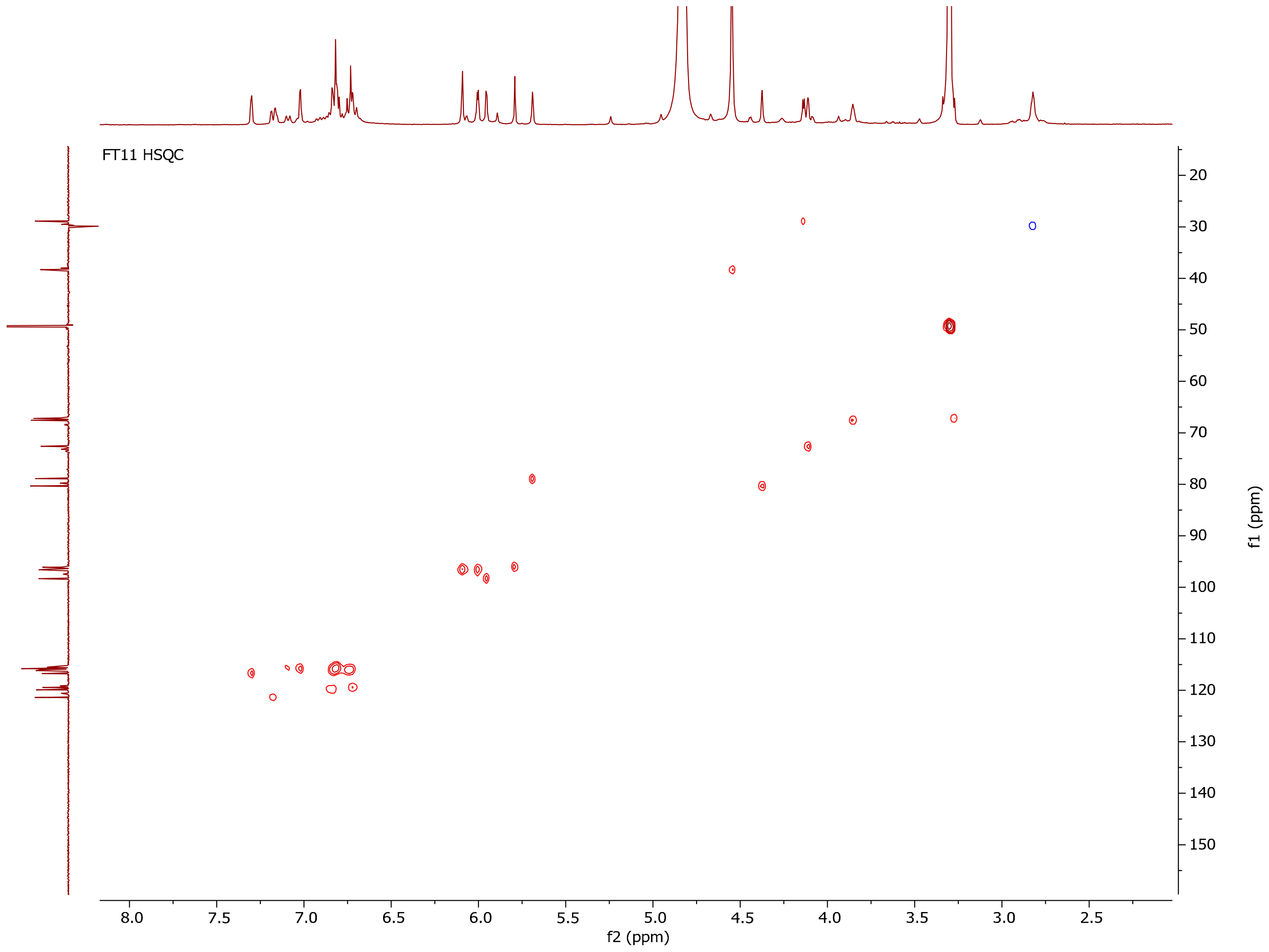

Figure S8. HSQC spectrum of compound 1

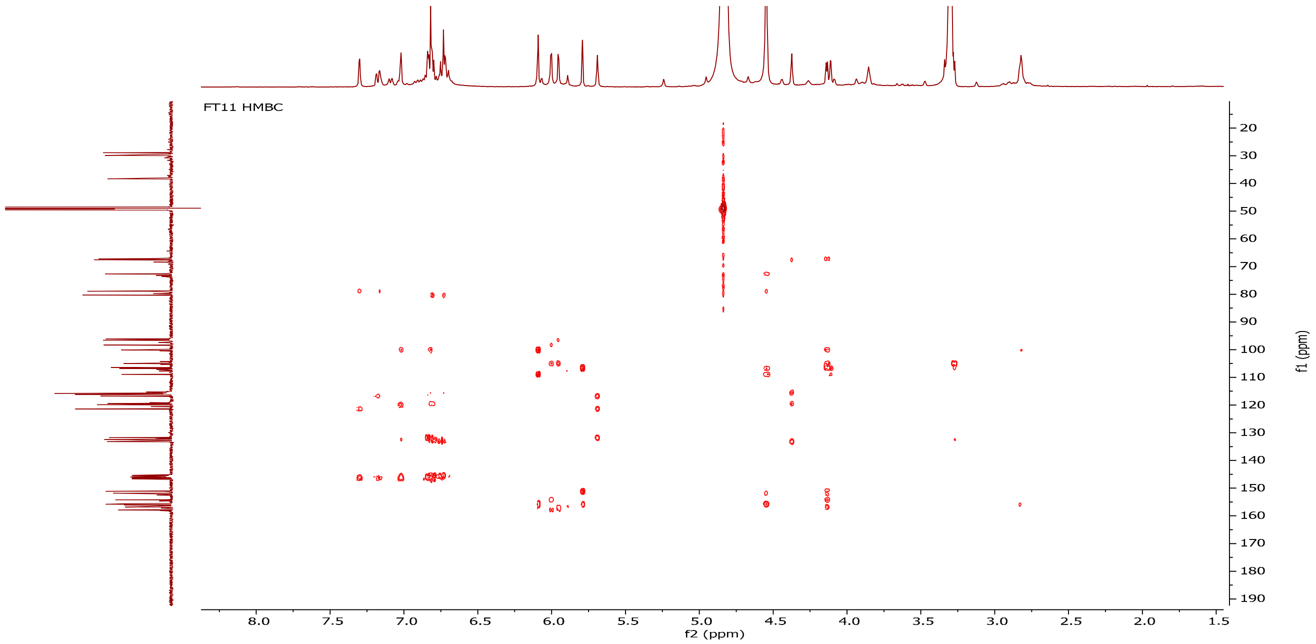

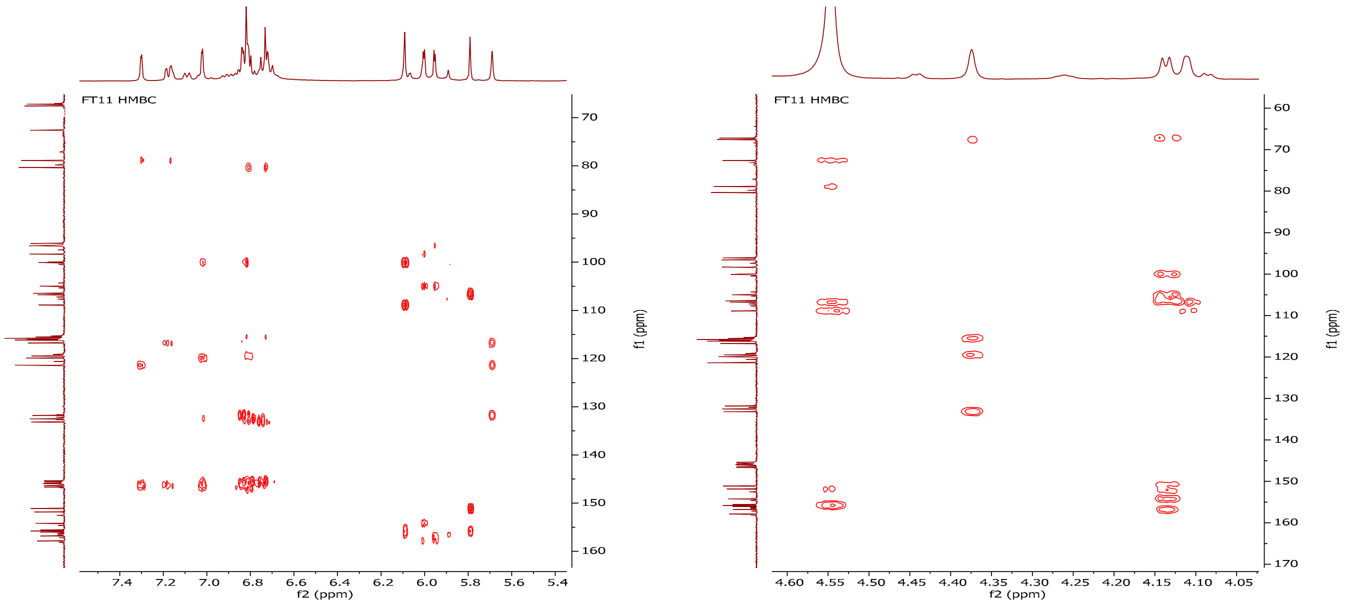

Figure S9. HMBC spectrum of compound 1
